# The prevalence of common mental disorders among Syrian refugees resettled in The Netherlands

**DOI:** 10.1192/j.eurpsy.2022.571

**Published:** 2022-09-01

**Authors:** M. Patanè, P. Cuijpers, A. De Graaff, R. Farell, M. Sijbrandij

**Affiliations:** Vrije Universiteit Amsterdam, Clinical, Neuro- And Developmental Psychology, Amsterdam, Netherlands

**Keywords:** Refugees, Epidemiology, Common mental disorders, Structured Clinical Interview

## Abstract

**Introduction:**

Refugees are at elevated risk of developing common mental disorders (CMD) as they may have been exposed to stressors and traumatic experiences before, during and after their movement. However, prevalence rates of CMDs among refugees reported across studies vary strongly.

**Objectives:**

To examine the prevalence of CMDs (PTSD, anxiety, depression and somatic disorder) among Syrian refugees in the Netherlands, and the diagnostic accuracy of self-reporting questionnaires in Arabic.

**Methods:**

A sample of N=1339 adult Syrian refugees was randomly selected from the Dutch national population registry. Participants were approached in December 2020-March 2021 to complete questionnaires on symptoms of PTSD (PCL-5), anxiety/depression (HSCL-25), and somatic disorder (SSS-8). After the survey, a sub-sample was invited for a clinical interview using the Structured Clinical Interview for DSM-5 (SCID-5) to enquire about the presence or absence of PTSD, anxiety, depression or somatic disorder.

**Results:**

In total, 407 participants (53.6% female, M age=34.2yrs, SD=14.1) completed the survey. The majority (65.9%) arrived in the Netherlands in 2015-2017. Using a cut-off of PCL-5 ³33, 75 participants (18.4%) reported probable PTSD. Using a cut-off of ³1.83 on the HSCL-25 depression subscale and ³1.75 on the anxiety subscale, 153 participants (37.6%) reported depression and 135 (33.2%) reported anxiety, and using a cut-off of ³12.0 on the SSS-8, 121 (29.8%) reported somatic complaints. A sub-sample of 214 participants (52.6%) were followed-up with the SCID-5. Psychometric properties will be presented.

**Conclusions:**

Syrian refugees in the Netherlands are at high risk for the development of a CMD. Implications, strengths and limitations of the study will be discussed.

**Disclosure:**

No significant relationships.

